# Knowledge and self-confidence of healthcare workers to perform transurethral catheterization: a matter deserving attention!

**DOI:** 10.1007/s00345-025-05677-3

**Published:** 2025-05-16

**Authors:** Gokhan Calik, Zeynep Bahadır, Berk Madendere, Ozgur Arikan, Vahit Guzelburc, Engin Evci, Suleyman Sami Cakir, Bulent Altay, Pilar Laguna, Mehmet Kocak, Selami Albayrak, Rahim Horuz, Kubilay Sabuncu, Mustafa Boz, Bulent Erkurt, Mohamad Aosama Alrifaai, Abdullah Al Chaabawi, Mahmoud Alrais, Ibrahim Abdi Ali, Shaban M. S. Ashour, Jean de la Rosette

**Affiliations:** 1 Department of Urology, Faculty of Medicine, Medipol Mega University Hospital, Istanbul Medipol University, Istanbul, Turkey; 2 International School of Medicine, Istanbul Medipol University, Istanbul, Turkey; 3 School of Medicine, Istanbul Medipol University, Istanbul, Turkey; 4 Department of Urology, Goztepe Prof. Dr. Suleyman Yalcin City Hospital, Istanbul, Turkey; 5 Istanbul Medipol University, Urology Clinic, Pendik Health Application and Research Center, Istanbul, Turkey; 6 Faculty of Medicine, Department of Urology, Istanbul Medipol University, Sefaköy Health Application and Research Center, Istanbul, Turkey; 7 Faculty of Medicine, Department of Urology, Istanbul Medipol University, Kosuyolu Medipol Hospital, Istanbul, Turkey; 8 Multi-Omics Design and Analysis Studio (MODAS-SABITA), Istanbul Medipol University, Istanbul, Turkey; 9 Bashkir State Medical University, Ufa, Russia; 10 Department of Urology and Guangdong Key Laboratory of Urology, The First Affiliated Hospital of Guangzhou Medical University, Guangzhou, Guangdong China

**Keywords:** Trauma, Complication, Transurethral catheterization, Female urethral catheterization, Male urethral catheterization, Training, Healthcare workers, Urethral stricture, Self confidence

## Abstract

**Background:**

Patients may suffer from the sequela of complicated transurethral catheterization (TUC) such as urethral injury, infection, and stricture formation. We assessed the self-confidence, knowledge, and experience of healthcare professionals performing TUC.

**Methods:**

A multi-center, prospective, cross-sectional questionnaire-based study was performed among healthcare workers from 5 university hospitals. Data was transferred to an online Data Management System and self-confidence, knowledge, and experience levels among different healthcare roles were compared.

**Findings:**

Of all 747 participants, 8% did not feel confident, had enough knowledge or preparation skills regarding TUC. 23% never asked for help while performing TUC whereas 42% always asked for assistance (p < 0.0001).

Healthcare roles did not differ statistically in terms of TUC knowledge and understanding. However, healthcare workers in surgical specialties felt more confident in their knowledge (29% vs 21%).

Confidence in male catheterization skills rated as ‘well’ and ‘very well’ were reported highest by paramedics (71%, 20%) followed by nurses (48%, 20%), physicians (53%, 30%) and residents (50%, 36%).

In the event of difficult catheterizations, physicians mostly preferred the assistance of “urologists and urology residents” (64%) while nurses mostly reached out to other nurses (39%). Paramedics were the least likely to ask for assistance (40%) followed by nurses (26%), doctors (24%), and residents (13%) (p < 0.0001).

**Conclusion:**

A significant proportion of healthcare workers do not have the necessary knowledge and understanding of TUC and do not feel confident in their catheterization and preparation skills when challenged by a difficult TUC, which requires the reassessment of the training programs pre- and post-graduation. This will facilitate and create a safer environment for both the patient and the healthcare professionals.

*Trial registry*: ClinicalTrials.gov NCT05334225

**Supplementary Information:**

The online version contains supplementary material available at 10.1007/s00345-025-05677-3.

## Introduction

Transurethral catheterization (TUC) is a medical procedure performed to drain the urinary bladder, monitor urinary output, relieve urinary retention, and help diagnose urinary problems such as urinary retention, urinary pressure, and other problems by obtaining sterile urinary samples. It is mostly carried out by nurses, paramedics, physicians, and residents-in-training [1].

It is reported that annually over 100 million TUCs are performed and approximately 200 transurethral catheters are used every minute [2]. Knowledge of the female and male genitourinary tract anatomy, use of lubricants and a diligent, stepwise approach of the healthcare worker safeguards a seamless introduction of TUCs [3].

Although the procedure is low in complexity, difficult catheterizations could lead to significant morbidity, induce urethral injury, and may eventually result in a urethral stricture. False passage or insufflation of the balloon of the catheter in the urethra are the most common reasons for urethral injuries. These injuries may require further surgical treatments that may additionally increase morbidity, result in long-term complications and overall increase in healthcare financial burden. A Urethral catheter injury incidence of 0.3–1.3% is reported and males are reportedly more prone to urethral catheter injuries due to male urethral anatomy [4, 5]. It goes beyond saying that a difficult catheterization can be traumatic to both patient and the performer. Therefore, the term “complicated catheterization” is an umbrella term used for TUCs that require medical response from board certified urologist and urology residents-in-training.

Data on the knowledge and self-confidence of healthcare workers to perform TUCs are scarce [1]. Cohen et al. revealed that a significant number of medical students do not feel confident in their procedural skills [6]. Learning procedural skills until recently often relied on a “see one, do one, teach one” principle [7, 8]. This signifies the importance of structured training protocols to ensure successful TUCs both for patient and the performer [9].

In this report, we present the initial data of our prospective multicentre cross-sectional study. We aim to highlight the current state of knowledge and experience, and self-confidence of various healthcare workers in performing TUCs. Additionally, we investigated types of resolutions followed by healthcare workers when facing complicated catheterizations.

## Material and methods

This is a prospective, multi-center, cross-sectional questionnaire-based study to evaluate healthcare workers’ self-reported confidence and knowledge in transurethral catheterization (TUC). The study was planned as the first part of a two-stage prospective training protocol conducted in five university hospitals. The study population included physicians, nurses, paramedics, and residents-in-training. Participation was voluntary, and all participants gave written consent. The questionnaire design was based on the comprehensive literature review and consisted of three parts: registration, participant data, and survey (Supplementary Form 1). The registration section included date of consent, center ID, participant first and last name. Even though the names of participants were collected to help the distribution and collection process, the names were coded and pseudonymised to protect the anonymity of the participants. The participant data part consisted of demographic data such as age, gender, position, department, years of experience and a question regarding prior experience about urethral catheterization. The survey part had nine questions to assess confidence, experience, and resolution strategies followed, and included an open-ended comments/suggestion section.

A board-certified urologist at each of the 5 university hospitals acted as the site coordinator for the study. They were responsible for distribution, collection, and safe handling of the questionnaires. A list of employees provided by each hospital was used to track the distribution and follow-up process. Since all employees were not physically present on the day of the survey distribution, a week’s time was allocated for the distribution of the surveys. After distribution, the participants were given two weeks to fill out the questionnaire. Two reminders were given during these two weeks to reduce the non-response bias. The completed surveys were sent to the primary investigation site, coded, and entered into the Data Management System (DMS). At completion of data entry, a comprehensive auditing was carried out through independent double-entry and data quality assessments.

### Statistical considerations

Categorical variables were described as frequencies and percentages, and continuous variables were presented as mean and standard deviation The association between categorical variables were investigated using Chi-Square Test for independence. TUC knowledge and confidence were compared by medical role (doctor, nurse, paramedic, resident), gender, whether being a surgery department, years of experience, and type of formal training received. Understanding TUC procedures was categorized as “understand/understand well” versus other lower understanding categories, and similarly confidence with TUC was categorized as “confident/very confident” versus other lower levels of confidences. Association of participant characteristics with these binary categorizations were summarized through odds ratios from Univariable Logistic Regression models. All analyses were conducted using SAS^®^ Version 9.4 (Cary, North Carolina, USA).

## Results

All active healthcare workers employed at the five university hospitals were eligible to participate in the study. Out of 846 healthcare workers, 747 participants filled out the survey (88.3% response rate).

265 (35.5%) males, with a median age of 27 (range 18–68, interquartile range 24–37) participated in the survey (Table 1). Of the participants, 184 (24.6%) were physicians, 430 (57.6%) nurses, 55 (7.4%) paramedics, and 78 (10.4%) residents-in-training. 547 participants (73.2%) were in surgical specialties and 188 (25.2%) worked in non-surgical specialties. 12 participants did not disclose whether or not they’re in a surgical department. 255 (34.1%) participants had 0–2 years of experience, while 153 (20.5%) had 3–5 years and 339 (45.4%) had 6 years or longer professional experience.

**Table 1:** Participant characteristics by medical role

	All		Role							
			Doctor		Nurse		Paramedic		Resident	
	N	Col %	N	Col %	N	Col %	N	Col %	N	Col %
All participants (n, row %)	747	100.00	184	24.63	430	57.56	55	7.36	78	10.44
Gender	482	64.52	63	34.24	357	83.02	29	52.73	33	42.31
Female										
Male	265	35.48	121	65.76	73	16.98	26	47.27	45	57.69
Years of experience	255	34.14	–	–	188	43.72	22	40.00	45	57.69
0–2 years experience										
3–5 years experience	153	20.48	3	1.63	108	25.12	14	25.45	28	35.90
6+ years experience	339	45.38	181	98.37	134	31.16	19	34.55	5	6.41
Department	12	1.61	–	–	12	2.79	–	–	–	–
Not specified										
Non-surgical	188	25.17	57	30.98	100	23.26	2	3.64	29	37.18
Surgical	547	73.23	127	69.02	318	73.95	53	96.36	49	62.82
Do you perform male or female urethral catheterization?	154	20.62	37	20.11	94	21.86	16	29.09	7	8.97
No										
Yes	593	79.38	147	79.89	336	78.14	39	70.91	71	91.03
Number of times you ever experienced a traumatic or complicated catheterization (bleeding, false passage, balloon inflated in urethra) in your entire career?	413	55.29	74	40.22	270	62.79	34	61.82	35	44.87
0										
1	144	19.28	30	16.30	87	20.23	8	14.55	19	24.36
2–5	134	17.94	48	26.09	54	12.56	11	20.00	21	26.92
6+	56	7.50	32	17.39	19	4.42	2	3.64	3	3.85
Did you ever have difficulties to catheterize and needed help?	187	25.03	45	24.46	110	25.58	22	40.00	10	12.82
No, never										
Yes, I asked a urologists/resident of urology for help	236	31.59	117	63.59	70	16.28	6	10.91	43	55.13
Yes, I asked another medical doctor colleague for help	127	17.00	17	9.24	79	18.37	13	23.64	18	23.08
Yes, I asked another nurse colleague for help	197	26.37	5	2.72	171	39.77	14	25.45	7	8.97
Do you perform catheterization all by yourself?	348	46.59	54	29.35	238	55.35	34	61.82	22	28.21
No, always with the support of a college										
Yes, always (100% of the time)	174	23.29	64	34.78	78	18.14	4	7.27	28	35.90
Yes, sometimes (< 50% of the time)	225	30.12	66	35.87	114	26.51	17	30.91	28	35.90
Any training during medical/nursing school?	185	24.77	59	32.07	108	25.12	10	18.18	8	10.26
No learning at medical/nurse school										
Yes, when I was learning at medical/nurse school	562	75.23	125	67.93	322	74.88	45	81.82	70	89.74
Any training while working in the hospital?	445	59.57	116	63.04	233	54.19	23	41.82	73	93.59
No training while working in the hospital										
Yes, while working in the hospital	302	40.43	68	36.96	197	45.81	32	58.18	5	6.41
Any training through a urology training program?	702	93.98	169	91.85	404	93.95	54	98.18	75	96.15
No urology training program										
Yes, I followed the urology training program	45	6.02	15	8.15	26	6.05	1	1.82	3	3.85
Any training at all?	739	98.93	183	99.46	424	98.60	54	98.18	78	100.00
At least some training										
No training	8	1.07	1	0.54	6	1.40	1	1.82	–	–

**Fig. 1:**
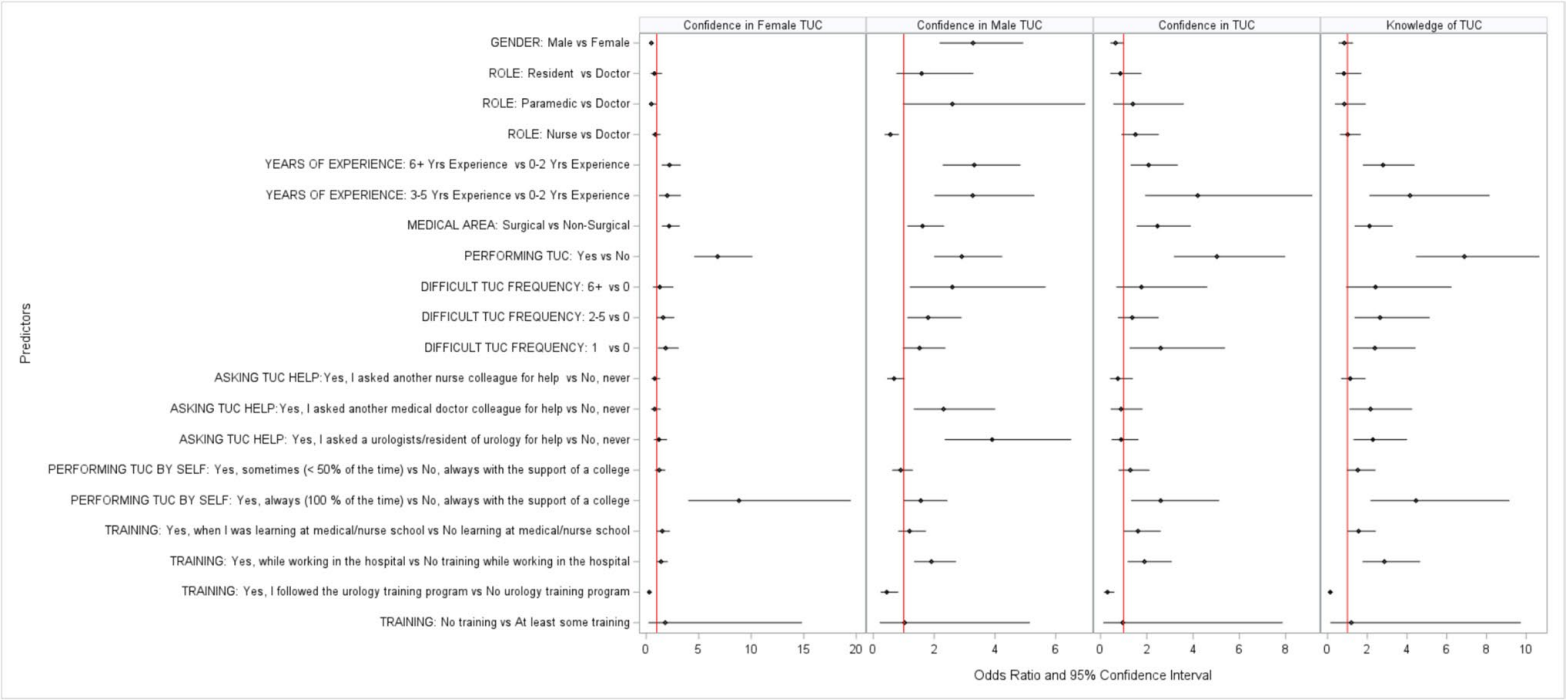
Factors associated with the knowledge of and confidence in TUC

Understanding and knowledge of TUC was not significantly different by gender (p = 0.42, medical profession (Minimum P-value > 0.60) (Fig. 1); however, as expected, those who reported longer years of experience reported higher level of knowledge as expected (p < 0.0001) and those in surgical areas reported higher level of understanding of TUC (p = 0.0008). The same conclusion was valid for the confidence with TUC overall, where the confidence in TUC increased with the years of experience and was higher for surgical departments.

While male participants expressed significantly higher confidence with male TUC (OR = 23.28, p < 0.0001), they expressed significantly lower confidence with female TUC compared to female participants (OR = 0.49, p = 0.0001). Those who work in surgical department reported higher confidence with both female and male TUCs compared to those working in non-surgical departments (OR = 1.62 and 2.18 with p = 0.01 and 00001, respectively). Those who had at least 3 years of experiences reported higher level of condience with male and female TUCs compared to those those with 2 or less years of experience (p < 0.01) as expected.

There was no significant difference between the doctors and residents in terms of confidence in male and female TUCs (p = 0.22 and 0.47, respectively), while paramedics reported higher confidence in male TUC (OR = 2.60, p = 0.057) and nurses reported lower confidence (OR = 0.56, p = 0.0052) and paramedics reported lower confidence in female TUC (OR = 0.49, p = 0.040).

Also 20.62% (154) of all participants didn’t perform female or male TUC. Those who perform TUC naturally reported higher level of knowledge and confidence (p < 0.0001). Those who have at least one difficult TUC had higher knowledge of TUC compared to those who did not perform any difficult TUC.

When facing difficulties performing TUC, physicians mostly(63.59%) opted for the help of “urologists and urology residents” while nurses mostly(39%) asked for the help of other nurses. Paramedics were less likely to ask for help of others (40%) than doctors(24.46%), nurses(25.58%) or residents(12.82%). Those who requested the help of urologists or other medical doctors in the event of a difficult TUC reported higher knowledge of TUC and higher confidence in male TUC compared to those who does not ask for help in a difficult TUC incident. Similarly, those who performs TUC by themselves < 50% of the time reported higher TUC knowledge and higher confidence in male TUCs compared to those who never performs TUCs by themselves.

185 (24,7%) participants reported no prior catheterization training in medical/nursing school.As expected, those with TUC training during medical school had higher level of TUC knowledge and higher confidence in TUC in general and male TUC while interestingly lower confidence in female TUCs compared to those who did not have any TUC training during medical school. Similarly, those with training during working in a hospital reported higher knowledge and understading across the board. Highly surprisingly, those who had TUC training in a urology program reported lower TUC knowledge and confidence compared to those who did not have such a training.

There appeared to be strong positive association between the years of practice and number of complicated catheterization (Spearman’s rank correlation = 0.29, p < 0.0001). There seems to be no association between training during medical/nursing school and the number of traumatic catheterization (Chi-Square p = 0.96).

Finally 47 out of 747 participants replied to the open-end question, and the most given comments were “Did not perform catheterisation ever or for a long time” (23 out of 47) and “because of the complexity/sterility of the catheterization process, I always perform it with a colleague” (11 out of 47).

## Discussion

A substantial number of healthcare workers do not feel knowledgeable and confident in their catheterization skills. Interestingly, paramedics were the most confident, while residents-in-training were the least confident. Nearly one quarter of healthcare workers never asked for assistance when performing catheterizations. However, almost half always requested help, usually from their peers, during the procedure.

TUC is a procedure that is widely performed in various medical settings such as emergencies, surgeries, and intensive or inpatient care. The procedure is usually performed by nurses, residents-in-training, paramedics and physicians. Even though our cross-sectional study showed no significant difference in knowledge and clinical skills related to TUC between different healthcare provider groups, confidence levels in performing TUC varied among the different healthcare provider groups.

Our study aligns with previous research highlighting knowledge gaps among healthcare workers when performing transurethral catheterization (TUC). Although we did not observe statistically significant differences in TUC knowledge between healthcare groups, 24.7% of participants reported having no prior TUC training in nursing or medical school. Moreover, those who received in-service TUC training felt more confident performing the procedure [10].

A systematic review by Alex et al. identified gaps in nurses’ educational preparation for indwelling catheter procedures [10]. Another study by Ozturk et al. found that supplementing traditional instruction with web-based TUC education improved nursing students’ knowledge and skills [11]. Cohen et al. also demonstrated that targeted TUC education increased medical students’ perceived usefulness of the training and desire for further instruction [6].

Together, these studies underscore the need for robust TUC training programs, which could include instructional videos, web-based modules, and simulation with augmented reality. Discrepancies in curricula and training opportunities across healthcare institutions likely contribute to variability in TUC knowledge and confidence between provider groups. Standardizing and improving TUC education across medical and nursing schools could help address these gaps. Our study reinforces the importance of targeted interventions to enhance healthcare workers’ preparation for safe and effective TUC placement. Lack of data regarding the knowledge and self-confidence of healthcare personnel that are performing TUCs on a regular basis warrants further investigation.

Our study found that healthcare professionals from different groups have varying preferences when seeking assistance for difficult transurethral catheterizations (TUCs). Physicians tended to request help from urologists, nurses from other nurses, and paramedics generally did not seek assistance during challenging catheterizations. We observed a trend of healthcare workers asking assistence to their peers if needed for help. This wide range of assistance preferences underscores the need for standardized protocols when difficult catheterizations occur. Implementing a formal policy could promote safer, more consistent patient care and prevent reliance on personal preferences and inconsistent care during challenging TUCs. It is also important to note that our study focuses on difficult catheterizations rather than traumatic catheterizations.

Implementing a protocol for quality assessment of transurethral catheterization (TUC) procedures in hospitals could help improve patient care. The protocol could involve tracking each catheterization complication in a logbook. This would allow for identification of any repeated catheterization failures for a given patient. The protocol could also implement a reward-based system for TUCs to incentivize careful technique. Under this system, a healthcare worker who has a significant number of repeated catheterization complications or failures could potentially lose the privilege of performing TUCs. In addition to restricting TUC privileges, the protocol could match more experienced healthcare workers with good quality assessment scores to high-risk patients in need of catheterization. These would include patients with strictures, BPH, Peyronie’s disease or on anticoagulants. Most importantly, the protocol could help identify the first responders, preferably urologist and urology residents-in-training, in cases of difficult catheterizations to standardize catheterization practices. The protocol should also include standardized intervention plans such as the use of flexible cystoscopy after certain numbers of TUC attempts and/or failed attempts at TUC. Implementing standardized quality assessment protocols for TUCs in hospitals can help protect both healthcare workers and patients from iatrogenic harm. The protocols promote accountability, experience-based assignments, and overall quality improvement of catheterization procedures.

In medicine, there is often a correlation between the number of times a procedure is performed (case volume) and the skill level of the healthcare workers performing it. Surprisingly, our study found statistically significant upward trend between healthcare workers’ years of experience and the number of traumatic urethral catheterizations (TUCs) they performed. However, it is highly likely that this correlation simply reflects the fact that more experienced workers perform more catheterizations overall. The ratio of total catheterizations performed to traumatic catheterizations for each healthcare worker could be a better indicator of skill level. Revising the number of TUCs to account for total experience would provide a more accurate picture of each worker’s proficiency with the procedure. On the contrary, these findings could be an implication of pseudo-confidence that gets generated with more experience which in return could increase traumatic outcomes in medical practices.

In addition, typically, female transurethral catheterization (TUC) is viewed as less challenging than male TUC. However, our study revealed that some participants still found female catheterization difficult. We also noted gender bias in the knowledge and self-confidence of healthcare workers when inserting a catheter in patients of the opposite sex.

### Strengths and limitations

Key strengths of this study are its large sample size and high response rate across a diverse sample of medical staff including residents, paramedics, physicians, and nurses from both academic and non-academic medical centers, thus providing heterogeneity.

However, the study was conducted among healthcare workers at only five hospitals. Thus, the results cannot be generalized beyond the respondents sampled. Additionally, the voluntary participation and the nature of survey-based studies introduce potential biases including voluntary response bias, recall bias, and selection bias due to lack of random selection. Reasons for non-participation are uncertain but may include various leaves of absence or unwillingness to participate. 

## Conclusion

TUC is a commonly performed procedure in medicine by healthcare workers. While considered a low complex intervention, a substantial number of healthcare personnel do not have the required knowledge and understanding of TUC and do not feel self-confident in their catheterization and preparation skills.

It is critical to implement more in-depth training programs to all healthcare workers pre- and post graduation.

Doing so could help provide enhanced patient care, lessen iatrogenic outcomes, and create a safer environment for both the patient and the healthcare worker.

## Supplementary Information

Below is the link to the electronic supplementary material.

Supplementary file1 (DOCX 37 KB)

## Data Availability

No datasets were generated or analysed during the current study.
