# Supplementary material for: Knowledge and self-confidence of healthcare workers to perform transurethral catheterization: a matter deserving attention!
Source: World J Urol. 2025 May 16;43(1):311. doi: 10.1007/s00345-025-05677-3 (PMC12084172; doi:10.1007/s00345-025-05677-3)
Supplement: Supplementary file 1 — Supplementary file1 (DOCX 37 KB) [file 345_2025_5677_MOESM1_ESM.docx]

**Knowledge and self-confidence of healthcare workers to perform transurethral catheterization: a matter deserving attention!**

Gokhan Calik^1,3^*, Zeynep Bahadır*^3^, Berk Madendere^3^*, Ozgur Arikan^4^, Vahit Guzelburc^1^, Engin Evci^5^, Suleyman Sami Cakir^6^, Bulent Altay^7^**,** Pilar Laguna^1,2^, Mehmet Kocak^8^, Selami Albayrak^1,3^, Rahim Horuz^1,3^, Kubilay Sabuncu^1,3^, Mustafa Boz^1,3^, Bulent Erkurt^2,7^, Mohamad Aosama Alrifaai^2^, Abdullah Al Chaabawi^2^, Mahmoud Alrais^2^, Ibrahim Abdi Ali^2^, Shaban M.S. Ashour^2^, Jean de la Rosette^1,2^

^1^ Department of Urology, Faculty of Medicine, Medipol Mega University Hospital, Istanbul Medipol

University, Istanbul, Turkiye

^2^International School of Medicine, Istanbul Medipol University, Istanbul, Türkiye

^3^School of Medicine, Istanbul Medipol University, Istanbul, Türkiye

^4^Goztepe Prof. Dr. Suleyman Yalcin City Hospital, Department of Urology, Istanbul, Türkiye

^5^Istanbul Medipol University, Urology Clinic, Pendik Health Application and Research Center, Istanbul, Türkiye

^6^Istanbul Medipol University, Faculty of Medicine, Dept. of Urology, Sefaköy Health Application and Research Center, Istanbul, Türkiye

^7^Istanbul Medipol University, Faculty of Medicine, Department of Urology, Kosuyolu Medipol Hospital, Istanbul, Türkiye

^8^Istanbul Medipol University, Department of Biostatistics and Medical Informatics, Istanbul, Türkiye

**REGISTRATION**

| Consent Date (dd-mm-yyyy): |  | | |
| --- | --- | --- | --- |
| First Name: |  | Last Name: |  |
| Participant ID: |  | Center ID: |  |
| PARTICIPANT | | | |
| Age (years): |  | | |
| Gender: | Male  Female | | |
| Role: | Nurse  Paramedic  Resident  Medical Doctor | | |
| Department: | \| General Surgery \| Urology \| \| --- \| --- \| \| Emergency Service \| Orthopedics \| \| Intensive Care \| Neurology \| \| Operating Room \| Internal Medicine \| \| Gynecology \| Cardiology \| \| Anesthesiology \| Other (Explain): \| | | |
| Years of Experience: | 0-2  3-5  6-10  11-15  16+ | | |
| Prior training about urethral catheterization: | Yes, when I was learning at medical/nursing school  Yes, while working in the hospital  No  Other (Explain): | | |

| **PRE-TRAINING SURVEY** | |
| --- | --- |
| Pre-training Survey Date (dd-mm-yyyy): |  |
| 1. Do you perform male or female urethral catheterization? | Yes  No |
| 1. How do you score yourself about your knowledge and understanding of urethral catheterization? | Absolutely no understanding  Understand a little  Understand well  Understand very well  Comments: |
| 1. Do you feel yourself confident about preparation for urethral catheterization including use of lubricant gel, cleaning, draping etc.? | I feel absolutely not confident  I feel a little confident  I feel confident  I feel very confident  Comments: |
| 1. Do you feel yourself confident about doing **male** urethral catheterization? | I feel absolutely not confident  I feel a little confident  I feel confident  I feel very confident  Comments: |
| 1. Do you feel yourself confident about doing **female** urethral catheterization? | I feel absolutely not confident  I feel a little confident  I feel confident  I feel very confident  Comments: |
| 1. Did you ever have difficulties to catheterize and needed help? | No, never  Yes, I asked another **nurse** colleague for help  Yes, I asked another **medical doctor** colleague for help  Yes, I asked a **urologists/resident of urology** for help  Comments: |
| 1. Do you perform catheterization all by yourself | Yes, sometimes (< 50% of the time)  Yes, always (100 % of the time)  No, always with the support of a college  Comments: |
| 1. Number of times you ever experienced a traumatic or complicated catheterization (bleeding, false passage, balloon inflated in urethra) in your entire career? | 0  1  2-5  6+  Comments: |
| 1. Comments/Suggestions |  |

**Table 2. Associations of Participant Characteristics with the Knowledge and and Confidence of TUC**

| **Independent Variables** | | **Confidence in TUC** | | **Confidence in Male TUC** | | **Confidence in Female TUC** | | **Knowledge of TUC** | |
| --- | --- | --- | --- | --- | --- | --- | --- | --- | --- |
|  |  | **OR (95% CI)** | **P-Value** | **OR (95% CI)** | **P-Value** | **OR (95% CI)** | **P-Value** | **OR (95% CI)** | **P-Value** |
| **Gender** | Male vs Female | 0.65 (0.42, 1.02) | 0.059 | 3.28 (2.18, 4.93) | <0.0001 | 0.49 (0.34, 0.70) | 0.0001 | 0.84 (0.55, 1.28) | 0.42 |
| **Role** | Nurse vs Doctor | 1.51 (0.90, 2.52) | 0.12 | 0.56 (0.37, 0.84) | 0.0052 | 0.87 (0.56, 1.36) | 0.55 | 1.01 (0.62, 1.67) | 0.95 |
|  | Paramedic vs Doctor | 1.40 (0.55, 3.60) | 0.48 | 2.60 (0.97, 6.97) | 0.057 | 0.49 (0.25, 0.97) | 0.040 | 0.84 (0.37, 1.92) | 0.68 |
|  | Resident vs Doctor | 0.86 (0.42, 1.77) | 0.68 | 1.59 (0.76, 3.29) | 0.22 | 0.78 (0.41, 1.51) | 0.47 | 0.82 (0.40, 1.70) | 0.60 |
| **Years of**  **Experience** | 3-5 Years vs 0-2 Years | 4.20 (1.93, 9.15) | 0.0003 | 3.27 (2.01, 5.30) | <0.0001 | 2.00 (1.21, 3.30) | 0.0068 | 4.15 (2.11, 8.16) | <0.0001 |
|  | 6+ Years vs 0-2 Years | 2.08 (1.30, 3.34) | 0.0024 | 3.32 (2.28, 4.84) | <0.0001 | 2.21 (1.48, 3.29) | 0.0001 | 2.79 (1.78, 4.38) | <0.0001 |
| **Department** | Surgical vs Non-Surgical | 2.46 (1.56, 3.90) | 0.0001 | 1.62 (1.12, 2.33) | 0.010 | 2.18 (1.49, 3.20) | 0.0001 | 2.11 (1.36, 3.28) | 0.0008 |
| **Performs TUC** | Yes vs. No | 5.03 (3.17, 7.98) | <0.0001 | 2.91 (2.00, 4.24) | <0.0001 | 6.8 (4.57, 10.12) | <0.0001 | 6.89 (4.45, 10.67) | <0.0001 |
| **Difficult TUC Frequency** | 1 vs. 0 | 2.60 (1.26, 5.38) | 0.010 | 1.52 (0.97, 2.37) | 0.066 | 1.85 (1.10, 3.09) | 0.020 | 2.38 (1.28, 4.43) | 0.0061 |
|  | 2-5 vs. 0 | 1.37 (0.75, 2.51) | 0.30 | 1.80 (1.12, 2.90) | 0.015 | 1.61 (0.96, 2.67) | 0.069 | 2.64 (1.36, 5.14) | 0.0041 |
|  | 6+ vs. 0 | 1.77 (0.68, 4.61) | 0.24 | 2.60 (1.20, 5.67) | 0.016 | 1.29 (0.64, 2.59) | 0.47 | 2.41 (0.93, 6.24) | 0.069 |
| **Difficult TUC help requests** | Urologists vs. None | 0.89 (0.48, 1.64) | 0.71 | 3.91 (2.35, 6.51) | <0.0001 | 1.21 (0.74, 1.98) | 0.45 | 2.28 (1.30, 4.00) | 0.0039 |
|  | Medical Doctor vs. None | 0.89 (0.44, 1.82) | 0.76 | 2.31 (1.33, 4.01) | 0.0029 | 0.80 (0.46, 1.38) | 0.42 | 2.16 (1.10, 4.26) | 0.026 |
|  | Nurse vs. None | 0.75 (0.41, 1.40) | 0.37 | 0.68 (0.45, 1.03) | 0.069 | 0.81 (0.50, 1.32) | 0.40 | 1.14 (0.68, 1.91) | 0.61 |
| **Performs TUC by self** | <50% of times vs. Never | 1.29 (0.78, 2.12) | 0.32 | 1.56 (1.00, 2.44) | 0.052 | 8.83 (4.00, 19.49) | <0.0001 | 4.45 (2.16, 9.16) | <0.0001 |
|  | 100% of times vs. Never | 1.62 (1.01, 2.60) | 0.046 | 0.90 (0.62, 1.30) | 0.57 | 1.23 (0.83, 1.82) | 0.30 | 1.52 (0.96, 2.41) | 0.076 |
| **Training in Med. School** | Yes vs. No | 1.62 (1.01, 2.60) | 0.046 | 1.19 (0.82, 1.73) | 0.36 | 1.53 (1.03, 2.26) | 0.034 | 1.56 (0.99, 2.43) | 0.048 |
| **Training in Hospital** | Yes vs. No | 1.90 (1.17, 3.08) | 0.010 | 1.91 (1.34, 2.72) | 0.0003 | 1.41 (0.97, 2.05) | 0.069 | 2.86 (1.76, 4.66) | <0.0001 |
| **Training in Urology Program** | Yes vs. No | 0.30 (0.15, 0.60) | 0.0007 | 0.44 (0.24, 0.82) | 0.0098 | 0.29 (0.16, 0.54) | 0.0001 | 0.13 (0.07, 0.25) | <0.0001 |
| **At Least some training** | No vs. Yes | 0.96 (0.12, 7.87) | 0.97 | 1.03 (0.21, 5.15) | 0.97 | 1.81 (0.22, 14.84) | 0.58 | 1.19 (0.14, 9.73) | 0.87 |
